# Perceptions and Experiences of Undergraduate Students Regarding Social Media as a Tool for Government COVID-19-Related Messages: A Qualitative Study in Nottingham, UK

**DOI:** 10.3390/ijerph20206903

**Published:** 2023-10-10

**Authors:** Sanvir Sandhu, Emma Wilson, Kaushik Chattopadhyay

**Affiliations:** 1 Nottingham Centre for Public Health and Epidemiology, School of Medicine, University of Nottingham, Nottingham NG7 2UH, UK; emma.wilson@nottingham.ac.uk (E.W.); kaushik.chattopadhyay@nottingham.ac.uk (K.C.); 2 Centre for Interprofessional Education and Learning, Faculty of Medicine and Health Sciences, University of Nottingham, Nottingham NG7 2UH, UK; 3 Nottingham Centre for Evidence Based Healthcare, University of Nottingham, Nottingham NG7 2RD, UK

**Keywords:** public health, social media, COVID-19, health promotion, e-health, digital health, health literacy

## Abstract

The rise of social media has given way to its use as a form of public health communication. Previous research has shown social media-based interventions to be effective, particularly for university students. Social media was used as a tool for public health messaging during the COVID-19 pandemic; however, there is a lack of information regarding the experiences and perceptions of such messaging and its effectiveness among UK university students. A qualitative study was conducted to explore the perceptions and experiences of public health messaging on social media during the pandemic, as well as the effects of such messaging. Fourteen semi-structured interviews with undergraduate students at the University of Nottingham, UK, were conducted. Five main themes were identified: effects of COVID-19 on university students, use of social media by university students, COVID-19 messaging on social media, effects of public health messaging on social media in compliance with COVID-19 guidelines, and improving public health messaging for the future. This study provides a novel insight into the experiences and perceptions of undergraduate students at a UK university regarding public health messaging on social media during the COVID-19 pandemic.

## 1. Introduction 

In early March 2020, the unprecedented impact of COVID-19 affected the entire global population, with widespread devastating consequences for health care, the economy, and society [1]. The UK government mandated a stay-at-home lockdown on 23 March 2020 [2], with a range of guidance published on the GOV.UK website.

Utilisation of social media platforms (e.g., Facebook, Instagram, and Twitter) allows information to be rapidly shared, and the rise of social media in the last two decades has changed and evolved the way in which individuals receive and share information [3], playing an imperative role in shaping the public’s perceptions of risk [4]. Social media has been identified as a useful tool for effective communication of emerging infectious diseases to the public, such as the use of Twitter during the Ebola outbreak in the UK [5] and increasing awareness of human immunodeficiency virus (HIV) testing and prevention services through social networks [6]. The UK government adopted similar methods throughout the COVID-19 pandemic [2] as a way of communicating guidelines to individuals. 

University students are highly active online users [7], and previous research has explored the role of social media interventions and behaviour change among university students and young people across topics such as sexual health and alcohol consumption [8,9]. As a result, technology-based interventions can be seen as a ubiquitous method for health promotion and education. At the beginning of the COVID-19 pandemic, UK universities were found to be more likely to post public health information and guidance related to COVID-19 if fellow institutions did, and those with larger student populations were likely to post information sooner [10]. A recent global study found young people to be the most active group online, demonstrating their high degree of digital literacy [11]. 

Using digital communication for health promotion has been shown to be effective and feasible. Social media has been used by governments across the world as a form of communication during the pandemic, with one example being Twitter [12], which was used by the UK government to disseminate public health information to a wide audience [13]. The credibility of healthcare professionals and scientific researchers was identified as being important in public health messaging [4], and social media has been shown to allow a high reach of messaging [14]. Users of social media during the pandemic were also shown to be more likely to make use of platforms to improve their health and wellbeing, demonstrating its usefulness in facilitating behaviour change [15]. Social media also helps to facilitate community engagement and allows individuals to be empowered to make their own health decisions [16]. 

It is important to note that social media can also act as a source of misinformation, as seen with vaccine misinformation and hesitancy during the pandemic [4,11,12]. While previous studies suggest that young adults are more likely to cross-reference such information [17], there is still a significant impact such misinformation has on the general population. 

Although public health communication via social media has been utilised during the pandemic, there remains a disparity in understanding the perceptions of university-age students on COVID-19 and the perceived effectiveness of such public health messaging. While cross-sectional research has been carried out in Canadian universities regarding social distancing messaging [18], there is a lack of primary research being conducted with university students in the UK [19], and no studies to date have explored the experiences and perceptions of undergraduate students regarding the use of social media during the COVID-19 pandemic. In addition, a recent systematic review indicated both the negative and positive impact of social media applications on public health communication, making note of how artificial intelligence can be used in both ways [20]. This demonstrates the need to further explore how to make better use of social media for public health communications.

The University of Nottingham is home to 46,000 students across three global campuses [21]. There is currently no research from this university regarding adherence to COVID-19 guidelines by students [22], and the availability of COVID-19 resources on the university’s website suggests that there is an opportunity to explore the effectiveness of this guidance on a large student population. 

This study aims to understand how undergraduate students at the University of Nottingham have used social media platforms during the COVID-19 pandemic and to explore the perceived effectiveness of both the government and the University of Nottingham’s public health messaging.

## 2. Materials and Methods 

This qualitative study was conducted in Nottingham, United Kingdom, from March to September 2022. The reporting of this study was guided by the JBI Critical Appraisal Checklist for Qualitative Research [23].

### 2.1. Study Design 

A qualitative study was carried out among undergraduate students at the University of Nottingham who were living in the UK when the COVID-19 pandemic emerged in early 2020. 

### 2.2. Sampling and Recruitment

Purposive sampling was used as the primary recruitment method for participants through self-selection. Individuals scanned a QR code on a recruitment poster, which directed them to submit a Microsoft Form. If individuals matched the inclusion criteria, the primary researcher (S.S.) then contacted them to arrange a time for an interview.

A total of 24 individuals expressed interest in the study; however, not all of these individuals matched the inclusion criteria of being an undergraduate student. A total of 14 individuals took part in the study. with a total of 14 eligible participants taking part in the study.

### 2.3. Data Collection

Semi-structured interviews were conducted by a trained qualitative researcher (S.S.). A pre-developed interview guide (see Appendix A) was used for all interviews, which took place in June and July 2022. Demographic information on age, gender, ethnicity, and university course was also obtained. Interviews were conducted in English via Microsoft Teams or telephone (as per participant preference). Interviews lasted between 21 and 45 min, with an average length of 29 min. All interviews were audio recorded with permission, and field notes were also obtained during the interview to pick up facial expressions and tone of voice that would not be visible post-transcription.

The first three transcripts were transcribed verbatim in order to become familiar with the data, and the remaining 11 recordings were transcribed by the university’s transcription service following completion of a privacy agreement. Any inaccuracies were corrected by listening to all recordings and making any necessary edits in order to ensure the transcripts were of high quality. All participant details were anonymised before being transcribed, including the removal of any identifiable information, and each transcript was assigned a number. Once data saturation was reached, no further interviews were conducted [24,25].

### 2.4. Data Analysis

Data from the interviews were analysed inductively through thematic analysis, using Braun and Clarke’s six stages as a guide [26,27]. NVIVO-12 (NVivo) software (Lumivero, Nottingham, UK) was used in conjunction with the six stages to generate codes, and an open coding approach was initially employed before progressing to axial coding in order to identify the most important and relevant codes [28]. S.S. acted as the primary instrument of analysis. Themes were then followed by a selective coding process, with NVivo being used to visualise the data. Final themes and subthemes were defined and named by all members of the team to ensure triangulation [29].

### 2.5. Ethical Considerations

Research ethics approval was obtained from the Faculty of Medicine and Health Sciences Ethics Committee at the University of Nottingham, with favourable opinion being received from the Faculty of Medicine and Health Sciences Ethics Committee on 25 April 2022. All participants were provided with the participant information sheet, which detailed the study’s aims and purpose, and all participants provided written informed consent prior to the interview. Participants were reimbursed with a £10 Amazon shopping voucher for their time.

### 2.6. Reflexivity

S.S. acting as the primary instrument of analysis in this study meant it was important to not let the researcher’s own philosophical worldview affect the research process [30]. S.S. was a current student at the time of research, and so it was important to ensure any preconceptions or thoughts were set aside.

The interview participants had no relationship with any members of the research team. Since COVID-19 is an issue that has affected everyone in different ways, it was imperative to stay neutral and account for emotional responses. To account for any biases that may have occurred throughout the process, the researcher engaged in bracketing to set aside assumptions and improve the rigour of the research [31]. 

## 3. Results

In total, 14 students took part in the study. Table 1 illustrates the demographic characteristics of the participants. 86% of participants were female, and the age range of 20–21 years old made up 58% of the sample. Half of the sample came from a White British background, with the other half being from ethnic minority backgrounds. Participants came from a variety of different academic backgrounds and across a range of academic years.

Initially, an open-coding approach was adopted following the transcription of all interviews. This included a broad range of codes, such as general observations on students’ general experience and wellbeing during the pandemic (e.g., feeling a sense of isolation and frustration at being left to ‘figure it out alone’), as well as their usage of social media in general, and other factors such as using TikTok more regularly than other platforms for public health communication. These initial codes were then assessed to draw connections, shifting to an axial coding approach and grouping them (e.g., establishing a link between negative and positive thoughts of social media as a public health messaging tool being part of the same theme). Axial coding was eventually adopted, resulting in the identification of final themes that aimed to answer the research aim.

The following five final themes were identified: (1) effects of COVID-19 on university students; (2) use of social media by university students; (3) COVID-19 messaging; (4) effects of public health messaging on social media in compliance with COVID-19 guidelines; and (5) improving public health messaging for the future.

### 3.1. Effects of COVID-19 on University Students 

Participants discussed how COVID-19 affected both their academic and social lives at university. Participants mentioned how they adjusted to the pandemic as well as their emotional response to COVID-19.

#### 3.1.1. Adjusting to the Pandemic

During the COVID-19 pandemic, many students struggled to adjust to the shift to online learning, and feelings of uncertainty were apparent.

“Yeah, it was quite a weird situation to be in, and obviously it was something that no one could kind of prepare you for. Everything was very up in the open. The only real information that we could get from people was through our peers, who also did not know anything that was going on.”

(English student, fourth year).

#### 3.1.2. Emotional Response to COVID-19

Strong feelings of isolation and loneliness were evident for university students during the pandemic, and this was perceived to have impacted their educational and social performance. Anxiety around ensuring family members were safe was also reported by participants.

“Pretty bad, to be honest. Obviously, yeah, I am a student nurse, so we kind of were thrown into a bit of a limbo … and it was kind of really weird … kind of lonely.”

(Adult Nursing student, third year).

### 3.2. Use of Social Media by University Students 

All study participants were active social media users, engaging with a wide variety of platforms. Students reported on both usage and public health messaging. 

#### 3.2.1. Social Media Usage

While all participants reported being active social media users, the average daily usage of social media platforms varied between participants. Social media usage increased during COVID-19-related lockdowns, often attributed to boredom and wanting to keep in touch with the wider world.

“I am sure it would have gone up. I do not know precisely, but I am sure I used it more just because there was more time being at home. And, you know, in between lectures or just having to stay at home during a lockdown, you end up finding yourself being on your phone all the time.”

(Medicine student, 4th year).

Many different platforms were used by participants, including Facebook, Instagram, Snapchat, TikTok, Twitter, and YouTube. Platforms such as Facebook and Snapchat were perceived more as communication tools, while Twitter was more likely to be used to source COVID-19 information.

“Twitter … one of the main sources of information in regard to having updates about COVID-19 at the time when it was like quite new and it was all foreign to us … it was just snippets of information that would be like easy to interpret.”

(Pharmacology student, 1st year).

#### 3.2.2. Social Media as a Public Health Messaging Tool

Participants viewed social media as a positive tool for communicating health information, with the high reach of such platforms being noted. However, participants also acknowledged the drawbacks of social media as a public health messaging tool, such as the dangers of misinformation. Many participants mentioned the importance of not using social media as a sole source of health information, and cross-referencing information with reliable news sources was expressed.

“I think it should never be the primary source of information because it is not accessible to quite a large group of people, but I do not see any harm in using it. Supplementary to like to support the messages that they are putting out on.”

(Mathematics student, 3rd year).

### 3.3. COVID-19 Messaging

COVID-19 public health messaging on both a government and University of Nottingham basis was explored, as well as the perceived effectiveness of these messaging tools. Participants also discussed some alternative sources of COVID-19 information.

#### 3.3.1. Government Public Health Messaging on Social Media

All participants recalled seeing some form of government public health messaging surrounding COVID-19 on social media at some point during the pandemic, in particular via Instagram and TikTok. Government-promoted posts were also commented on, such as the ‘hands face space’ slogan and the uptake of the COVID-19 vaccine. Students expressed the importance of sharing these messages to gain maximum impact.

“So like on people stories, it was like a stay-at-home message and they have come up highlighted if they involved something to do with COVID like at the top.”

(Medical Physiology and Therapeutics student, 1st year).

#### 3.3.2. Perceived Effectiveness of Government Messaging

There was a general consensus among participants that government messaging was effective and frequent at the height of the pandemic on social media, but a decline was evident over time. Government messaging was also viewed as being easily misconstrued and open to interpretation.

“So, at the beginning it was very, very easy to constantly have information, but as we moved on, I feel like it got a bit less effective.”

(English student, 4th year).

While the repetitive messaging was seen as helping to keep COVID-19 guidelines at the forefront of the public’s minds, it was also acknowledged that the government was ineffective in combatting misinformation. Students did empathise with the unprecedented nature of the COVID-19 pandemic; however, the real-time ability to compare the UK government’s messaging with other countries meant that it was easy to see how the UK government could have improved things, as well as observing a lack of distinct messaging for minority groups.

“I think there was quite a heavy reliance on social media, and it did not reach a lot of kind of minority groups; they did not get vaccinated because of a lack of messaging, and they just did not have as much access.”

(Geography student, 3rd year).

#### 3.3.3. Communications from the University of Nottingham

Students mentioned receiving regular emails related to COVID-19 from the University of Nottingham at the beginning of the pandemic. COVID-19 information was also described as being physically present on the university campus, such as signage on social distancing, mask wearing, and sanitising hands. Those students who followed the university on social media recalled also seeing information on these platforms, as well as on the MyNottingham app. In contrast, some students did not follow the university on any social media platforms and therefore did not engage with this messaging.

“I think most of it was emails, if not like face-to-face and with like posters and that kind of thing. But if it was online, it was mostly through emails, yeah, or on their Instagram.”

(Medical Physiology and Therapeutics student, 1st year).

#### 3.3.4. Perceived Effectiveness of the University of Nottingham

Many students felt that the university handled the transmission of COVID-19 information well for the most part and was effective in reaching students quickly, although this was seen to vary between faculty and/or departments. A lack of justification for some university decisions was perceived by some students, and social media content was seen to be a replica of email content.

“I think we’ve got a good amount of information through emails, and if anything, I had too many emails.”

(Medicine student, 4th year).

#### 3.3.5. Other Sources of COVID-19 Information

In addition to the government and the University of Nottingham, participants expressed receiving COVID-19 information from other sources. These included news sources and websites, posts from social media influencers on Instagram, personal stories of family members, and light-hearted content such as memes on TikTok and YouTube videos.

“Yeah, and then on Twitter there was loads on like World Health Organisation and political figures and public figures…And then Instagram. I saw a lot of just like sort of public individuals…I think I remember in the first lockdown there was a big like push online by influencers saying you know like stay at home and things like that.”

(Geography student, 2nd year).

### 3.4. Effects of Public Health Messaging on Compliance with COVID-19 Guidelines

The effects of public health messaging on individuals’ behaviour were discussed by participants, as well as changes in messaging over time and a lack of clarity regarding guidelines.

#### 3.4.1. Effects on COVID-19 Behaviour

Many participants explained how they felt that they would have followed COVID-19 guidelines regardless, but engaging with government messaging may have subconsciously helped to remind them. The possibility of individuals becoming desensitised to such messaging over time was also acknowledged.

“Subconsciously, it probably did, and I remember those things, so it probably did influence me kind of like subconsciously.”

(Adult Nursing student, 3rd year).

#### 3.4.2. Changes over Time

The changes in COVID-19 government guidelines were reflected in messaging, and many participants acknowledged this. Gradual changes in messaging as restrictions eased were noted, with some messaging still remaining despite now feeling redundant. The content of the messaging was viewed as having shifted from social distancing measures to promoting vaccination uptake over time.

“It all seems like everything’s been put up and left there and people just ignore it and also all the hand sanitising things.”

(French Studies, 2nd year).

#### 3.4.3. Lack of Clarity

Despite a high presence on social media, students expressed that the government’s messaging felt inconsistent at times, such as sudden changes in COVID-19 guidelines not being communicated properly. In addition, students felt that the University of Nottingham was inconsistent in mandating rules on household mixing between halls of residence, and help with adjusting to online learning was also seen as inconsistent.

“I think maybe if advertising had been a bit more consistent, people might have been more likely to adhere to guidelines because as it went on people stuck to them less and less.”

(Psychology and Cognitive Neuroscience student, 1st year).

### 3.5. Improving Public Health Messaging for the Future

Participants suggested ways in which the government and University of Nottingham could improve their messaging in the future, as well as highlighting specific public health areas related to students.

#### 3.5.1. Improving Government Public Health Messaging

Inclusion of tailored messaging, real-time updates, and utilising more platforms were viewed by participants as ways in which the government could have improved their messaging during the COVID-19 pandemic. Incorporating clearer language was also seen as an important point, as was considering minority groups and catering to the young demographic. Instagram was viewed as the best platform to circulate public health information by the government, and TikTok was also seen as being useful. The missed opportunity for the government to make use of an increased number of people staying at home through health promotion initiatives was also discussed.

“Everyone was in the house for months on end. They could have easily tried and gotten a good HIV testing programme… Yeah, like it is just a prick test and you can do it in your house.”

(Medicine student, 3rd year).

#### 3.5.2. Improving University of Nottingham Public Health Messaging

Informing students about changes in university regulations and taking an educational standpoint throughout the pandemic was viewed as an important point for the future by participants. Other suggestions to enhance public health messaging from the university included information on where to get vaccinated, facilitating first-year group chats on social media, providing more welfare support, distributing face masks around campus, and setting up a dedicated COVID-19 page on social media and the Moodle platform.

“I think if we were to have a second COVID outbreak, it is just to make sure that there are regular updates with students so they know what is going to happen with them.”

(English student, 4th year).

#### 3.5.3. Key Public Health Areas for the Student Population

Participants discussed a wide range of public health areas relevant to the student population for future promotion on social media. These included mental health, sexual health, alcohol/substance misuse, nutrition, domestic violence, cancer screening, as well as freshers’ flu and other communicable diseases. Providing key information and signposting to services such as sexual health clinics and wellbeing programmes were some of the ways in which participants believed this could be carried out. Social media was viewed by participants as the best way to relay these health messages, such as the creation of Instagram videos by the University of Nottingham on these issues and utilising societies and sports groups to spread information. 

## 4. Discussion 

This qualitative research study has found that university students engage with social media as a form of public health messaging and receive information about COVID-19 during the pandemic through social media. This study is the first known qualitative study to explore the perceptions and experiences of undergraduate students at a single UK-based university regarding the use of social media as a tool for public health messaging during the COVID-19 pandemic.

Many students were found to be regular users of multiple social media platforms, in line with previous literature [32,33,34]. Social media was highlighted as a primary or secondary source of information for students during the pandemic, and this was shown to have increased over time. The emerging use of TikTok as a viable health information platform for young adults [35] was also evident; however, there is a lack of research into the use of such new platforms for health information, and further studies are warranted.

Social media platforms have been shown to provide an opportunity for misinformation to be rapidly spread due to a lack of verification or moderation [35,36], and this study parallels such findings by indicating that social media is not the primary source of information for most students [10]. Going forward, it is important that governments and organisations look at how best to use social media to increase impact and widen reach by ensuring validation of information as well as utilising relatively novel strategies and platforms.

This study goes beyond previous research into COVID-19 and social media by highlighting the use of all platforms by the UK government over the course of the pandemic, rather than one platform in particular [37]. Engagement with COVID-19 information on social media regularly helped to keep messages at the forefront of students’ minds, and previous studies indicate how social media is effective for young people regarding behaviour change [8,9,38,39].

This research provides evidence for the use of social media in health promotion in relation to COVID-19, as it allows individuals to empower themselves to reduce their risk of contracting COVID-19 through taking precautionary actions. Previous research has found social media to be a credible tool for health promotion [40], and this study adds to the literature by providing the perspectives and experiences of students during an unprecedented pandemic that heavily involved online communications. In conjunction with the health belief model [41], social media can act as a way of promoting healthy behaviours related to COVID-19 for university students whose perceived susceptibility and severity of contracting the COVID-19 virus have been heightened at the beginning of the pandemic. Students carried out cues to action, such as staying at home during national lockdowns. The concept of self-efficacy is integral to the model [42], and social media messaging would have allowed individuals to believe they were able to carry out such behaviours due to the effectiveness of messaging on social media. 

Behaviour change is integral to health promotion measures such as adherence to COVID-19 guidelines, with social learning theory playing a role in this [43]. In this case, students who observe their peers or academic staff exhibiting positive behaviours attributed to COVID-19 guidelines would be more likely to model such behaviour as a result of environmental stimuli [44]. The application of social learning theory in health promotion has been studied previously [44,45,46], and this research adds to this evidence base by demonstrating how students were more likely to adhere to guidelines if they had seen other people doing so, both online and offline.

This study goes beyond previous research exploring the inconsistency of government messaging by exploring this among university students, a population more likely than other age groups to engage with social media and change their behaviour. In addition, it was mentioned that the government’s messaging was not always inclusive of minority groups. This has been reflected in the media, such as the production of a video widespread on social media platforms by the government encouraging individuals from ethnic minority groups to get vaccinated, following high vaccine hesitancy and misinformation in these groups [47]. Future public health messaging by the government should be inclusive and diverse from the very beginning to prevent similar occurrences, and social media can be used as a platform within these communities [48].

This study is unique in focusing on a single university in the UK regarding COVID-19 communications. Regular email communication from the university being seen as an effective method by students contrasts previous views suggesting students are more likely to use social media for information from their university provider compared with more traditional email formats. This is further amplified by some participants mentioning how they do not even follow the University of Nottingham on social media platforms, indicating a need for the university to work on promoting their platforms to improve engagement in the future.

Students mentioned feelings of isolation and anxiety during the COVID-19 pandemics. This echoes that of the general population, such as in a cross-sectional study identifying that higher social media consumption was associated with higher levels of anxiety and depression [49]. While this research does not explore the effects of COVID-19 on mental health explicitly, the feelings of anxiety and loneliness mentioned by participants in relation to the university’s online teaching and student accommodation provision suggest that students may not perceive the University of Nottingham to have effectively handled the pandemic well. Previous research has found the use of Twitter to be effective for mental health [50], therefore highlighting that similar initiatives could be used by the University of Nottingham to improve student wellbeing.

Students mentioned a wide range of potential public health issues for future discussion, including mental health, sexual health, and excessive alcohol consumption. Notably, students also mentioned topics such as freshers’ flu and early awareness of the importance of cancer screening, which have not been extensively studied in university populations as of yet. Using social media platforms to engage with students is imperative to the success of future public health messaging and should be regarded as a highly important area to utilise and invest in for future interventions.

### Study Strengths and Limitations

A major strength of this study is that it used qualitative methodology in order to obtain detailed, rich data on a topic with a lack of qualitative exploration. Furthermore, the study used the ‘gold-standard’ thematic form of analysis in health research [51].

Employing a purposive sampling technique ensured that the study population was not predetermined and allowed for a range of individuals from different backgrounds to be included [52]. This increases the transferability of the research findings. In addition, data saturation was reached in this study, showing that an acceptable sample size was achieved [24].

Although purposive sampling lends itself well to representing the wider population, this study only included two male participants. Men are often underrepresented in studies regarding social media [53], and so a different sampling technique (e.g., systematic sampling) may have overcome this.

Selection bias may have occurred in this study since recruitment posters were circulated around specific locations of the university campus, which would only reach students who visited these areas. The study was limited to current undergraduates at the University of Nottingham, meaning their experiences would have been specific to one context and so may reduce the transferability of findings to other universities in the UK.

Social desirability bias may have limited the generalisability of the findings [54]. The researcher took steps throughout the process to mitigate against this, such as encouraging participants to speak freely and reminding them their responses remain anonymous and confidential at the beginning of the interview.

Participants were provided with a £10 Amazon shopping voucher following the interview, and incentivisation can be seen to alter risk perception by participants [55]. However, the provision of a participant information sheet and consent form mitigated against this.

## 5. Conclusions

University students are avid, regular social media users, and this usage increased during the COVID-19 pandemic. Many students felt that, despite government messaging during the COVID-19 pandemic being visible across different platforms, it was easily misconstrued and open to interpretation. Feelings of inconsistency and a lack of transparency were also highlighted by participants, which is in line with the feelings of the general population. University of Nottingham students generally spoke positively of their university’s ability to communicate information on COVID-19, although it was noted that certain guidelines sometimes lacked further explanation or clarification from the university.

The findings of this study and the identification of distinct themes help to gain a better understanding of the usage of social media platforms by university students during the COVID-19 pandemic, as well as their perceived effectiveness. It is hoped that the findings will add to the literature and improve public health messaging on social media as we continue to live with COVID-19, as well as enhance messaging across a range of other public health issues that primarily affect student populations.

## Figures and Tables

**Table 1:** Participant characteristics.

	Gender	Age Group	Ethnicity	Year of Study at University	Course Studied
P01	Female	20–21	White British	first	Psychology and Cognitive Neuroscience
P02	Male	18–19	Black British	first	Pharmacology
P03	Female	20–21	White British	fourth	Nursing (Adult)
P04	Female	18–19	Asian British	first	Pharmacy
P05	Female	20–21	White British	second	Geography
P06	Female	18–19	White British	first	Medical Physiology and Therapeutics
P07	Female	20–21	White British	fourth	English
P08	Female	20–21	White British	third	Geography
P09	Female	22–23	Asian British	third	Medicine
P10	Female	20–21	Mixed	second	French Studies
P11	Female	22–23	Asian	third	Pharmacy
P12	Female	20–21	White British	third	Mathematics
P13	Male	22–23	Other	fourth	Medicine
P14	Female	20–21	Asian	third	Medicine

## Data Availability

The data analysed in this study can be accessed by emailing the authors on request.

## Appendix A

- Start off by introducing yourself and thanking the participant for agreeing to talk.
- State title of study: This research is looking at the perceptions and experiences of undergraduate students at the University of Nottingham regarding the use of social media as a tool for government public health messaging during the COVID-19 pandemic.
- Mention how research aims to find out more about the experiences, thoughts, and opinions of students in regard to social media and public health messaging. And it is hoped that this research will help shape future public health messaging across other areas for students.
- “Everything you say in this interview will be kept confidential. If you mention the names of anybody else, we will remove these names from the transcript after it is finished, so please feel free to speak as freely as possible! The data from this interview will be anonymised and pooled, and we will look to draw a consensus of opinion. You have the right to withdraw from this study at any point. The interview will be recorded.”
- Do you have any questions?
- Before we commence, are you happy to give your consent to take part in this study?

1. Social media use and experience as a student during the pandemic
  (a) First of all, I will start off by asking: What was your experience as a student during the COVID-19 pandemic?
  (b) As I said previously, this study is looking at the use of social media as a form of public health messaging during the pandemic. Tell me about your typical social media use on a day-to-day basis?
  (c) Which social media platforms do you use on a regular basis?
  (d) Going back to thinking about the first lockdown in March 2020, can you tell me about your use of social media platforms during that time?

2. Government messaging on COVID-19
  (a) In regard to COVID-19 information specifically, could you please tell me about the government public health messages you saw on social media regarding COVID-19 guidelines?
  (b) Do you think seeing these messages on social media had an impact on reminding you to follow such guidelines?
  (c) Did you see more or less government messaging on social media as time went on after the first lockdown and restrictions started to ease?
  (d) Do you remember seeing anything about COVID-19 on social media from a source other than the government?
  (e) Now that we are over 2 years into the pandemic, what are you currently seeing on social media in the last few months regarding COVID-19? *(Include point-reminders such as social media now, social media in the past, and following guidelines).*
  (f) Please tell me how effective you think the government was in using social media during the pandemic?

3. UoN messaging and COVID-19
  (a) You have told me what you think about the government and public health messaging; now we will talk a bit about the University of Nottingham.
  (b) Please tell me about the information you received on COVID-19 from the university?
  (c) Have you seen any guidance around campus or online about COVID-19, and what have you seen?
  (d) In a hypothetical situation, what do you think we could have done better as a university during the pandemic, from a social media perspective?
  (e) Did you feel that information on what to do in relation to the COVID-19 guidelines as a student was communicated effectively, both by the government and the university?

4. General thoughts and future recommendations
  (a) What are your general thoughts on the use of social media for public health messaging?
  (b) How would you like to see social media used in the future by the government for public health messaging?
  (c) Please tell me about any university-specific public health issues you would like to see talked about in the future on social media?

- Is there anything else you would like to add that we have not talked about?
- Thank the participant for their time. Mention the Amazon voucher to be emailed to the email address provided as a way of saying thank you.
- End on a positive note: “Thanks again; see you later!”
